# The 6^th^ international conference on envenomation by Snakebites and Scorpion Stings in Africa: a crucial step for the management of envenomation

**DOI:** 10.1186/s40409-016-0062-y

**Published:** 2016-03-16

**Authors:** Jean-Philippe Chippaux, Marc Hermann Akaffou, Bernard Kouadio Allali, Mireille Dosso, Achille Massougbodji, Benedito Barraviera

**Affiliations:** CERPAGE, Faculté des Sciences de la Santé, Université d’Abomey-Calavi, Cotonou, Bénin; UMR 216, MERIT, Institut de Recherche pour le Développement et Université Paris Descartes, Sorbonne Paris Cité, Faculté de Pharmacie, Paris, France; Institut Pasteur de Côte d’Ivoire, Unité d’Entomologie/Herpétologie, Abidjan, Côte d’Ivoire; Center for the Study of Venoms and Venomous Animals (CEVAP), São Paulo State University (UNESP – Univ Estadual Paulista), Botucatu, SP Brazil

**Keywords:** Envenomation, Antivenom, Epidemiology, Drug policy, Africa

## Abstract

During the 6^th^ International Conference on Envenomation by Snakebites and Scorpion Stings in Africa held in Abidjan, from 1 to 5 June 2015, the measures for the management of envenomation were discussed and new recommendations were adopted by the participants. The high incidence and severity of this affliction were confirmed by several studies conducted in African countries. The poor availability of antivenom, particularly because of the cost, was also highlighted. Some experiences have been reported, mainly those regarding the financial support of antivenom in Burkina Faso (more than 90 %) and Togo (up to 60 %) or the mandatory reporting of cases in Cameroon. Key recommendations concerned: improvement of epidemiological information based on case collection; training of health workers in the management of envenomation; policy to promote the use of effective and safe antivenom; and antivenom funding by sharing its costs with stakeholders in order to improve antivenom accessibility for low-income patients.

## Introduction

Scorpion stings in North Africa and snakebites in sub-Saharan Africa are responsible, respectively, for 750,000 cases of envenomation with 1,700 deaths, and 320,000 envenomations including ten thousands of deaths and much debilitating sequelae [1, 2]. Envenomation usually affects rural populations, generally young farmers whose income is low. Household surveys found that almost all victims of stings or bites by venomous animals were initially assisted by a traditional healer – out of those, more than a half were treated only by him – causing a consultation delay, which was detrimental to the clinical course.

Organized jointly by the Société Africaine de Venimologie – SAV (African Society of Venomology) and the Pasteur Institute of Cote d’Ivoire, the 6^th^ International Conference on Envenomation by Snakebites and Scorpion Stings in Africa (6^ème^ Conférence Internationale sur les Envenimations par Morsures de Serpent et Piqûres de Scorpions en Afrique) was held from 1 to 5 June 2015 at the Faculty of Medical Sciences, University Félix Houphouët-Boigny, in Abidjan, Côte d’Ivoire. As in previous meetings, this conference consisted of three different sessions: a two-day workshop on the management of envenomation; the scientific conference, which also extended over two days; and stakeholder meeting to discuss the availability of antivenoms (www.sav-asv.com/).

### Workshop on the management of envenomation

The first day of training involved forty trainers, mainly Ivoirians, to whom the methodological basis of venomology was presented (herpetology, epidemiology, biochemistry and toxicology of venoms, clinical manifestations and treatment of envenomation) in order to clarify the causes and consequences of the encounter between a human and a venomous animal.

The second day was dedicated to trainees and people that deal with envenomation: physicians, pharmacists, nurses, firefighters, rescue workers, paramedics and traditional healers. Over 200 people attended the course that explained about the circumstances, symptoms and treatment of snakebite in Côte d’Ivoire. The different clinical presentations, as well as the therapeutic approach have been described. A simple diagnostic and treatment algorithm was presented.

### Scientific conference

This session brought together about 200 participants from 18 countries from all continents (Germany, Belgium, Benin, Burkina Faso, Cameroon, Côte d’Ivoire, France, Ghana, Guinea, India, Kenya, Mali, Mexico, Morocco, Nigeria, Senegal, Switzerland, and Togo). Representatives of several countries, already registered (Algeria, Angola, Brazil, Congo, Democratic Congo, USA, Great Britain, Italy, Mauritania, Niger, Chad, Tunisia), could not attend, because unavailable or, most often, due to financial reasons.

In his inaugural lecture, Prof Abdulrazaq Habib presented an economic model showing the particularly profitable cost-benefit relation of antivenom use. Despite its high cost, the burden for public finances and society are greater when antivenom is not used taking into account the reduction of life expectancy, disability-adjusted life year (DALY) and quality-adjusted life year (QALY). The costs per DALY and averted deaths vary, respectively, from US$ 2,000 to US$ 6,000 and from US$ 100 to US$ 300, depending on the country.

Most studies confirmed the high incidence and severity of envenomation, and serious management deficiencies related in particular to late consultation, inaccessibility of antivenom and lack of training of medical personnel. Various communications dealt with the diagnosis of envenomation and treatment either by antivenom or herbal medicine. Overall, the availability of antivenom is inadequate, which was observed in most countries.

In this minicollection “Strategies for Management of Snakebites in Africa”, four studies selected among those presented at the conference are to be published in the *Journal of Venomous Animals and Toxins including Tropical Diseases* (JVATiTD):

- A research indicates that although the number of reported victims of scorpions stings is elevated in Morocco, snakebites also comprise a public health problem in the country that affects several hundreds of people, including some severe envenomation cases [3].
- Another study reports that in the region of Kedougou (eastern Senegal), the annual incidence is about 315 snakebites per 100,000 population. Mortality exceeds eight deaths per 100,000 inhabitants according to household surveys, whereas official health statistics reports less than a third of those [4].
- In Burkina Faso, more than 35,000 envenomation cases are notified annually with an average of 275 deaths. Despite these high numbers, a study reveals that only 1,150 doses of antivenom are administered every year in the region [5]. Therefore, although antivenom is imported from neighboring countries, the therapeutic coverage is far from enough.
- The other work revealed that in Benin, ultrasonography comprises a valuable tool that helps in the diagnosis and management of hemorrhagic disorders provoked by *Echis ocellatus* bites that represent more than 70 % of envenomation cases in savannas of sub-Saharan Africa [6].

During the general assembly of the African Society of Venomology, the creation of national subsidiaries – designed to relay the recommendations of SAV and facilitate their implementation –that favor management autonomy was unanimously approved.

### Stakeholders meeting

On the last day, an open discussion was held among stakeholders who were willing to participate. Once again, with the notable exception of the 4^th^ Conference in Dakar, in which the World Health Organization (WHO) was represented, international agencies, albeit invited, did not attend.

The experiences of several represented countries were exposed. Burkina Faso subsidizes antivenom price (up to 90 %), causing the retail price to be 2,500 FCFA (about US$ 5). Since the beginning of 2015, Cameroon introduced mandatory reporting of envenomation, as recommended by WHO. Senegal forced every pharmacy in the country to stock permanently at least one vial of antivenom. Togo has been supporting for five years the price of antivenom by 60 % in the public drug distribution system. Finally, Côte d’Ivoire has introduced the treatment of envenomation in the National Program of Universal Health Coverage that would be active by the end of 2015.

Following the debates, four major recommendations were unanimously adopted.

### Recommendations

1. **Epidemiological studies** should be performed to assess the therapeutic needs, particularly the amount of antivenom required and where it should be available. Health authorities in each country were encouraged to establish, as soon as possible, the mandatory reporting of envenomation.
2. **Training in the management of envenomation** should be restored rapidly in medical, pharmacy, and nurse schools. Meanwhile, training of health personnel in the diagnosis of envenomation and use of antivenoms should be organized within each country.
3. **Drug policy for antivenoms** should be adapted to the national context. Antivenom selection and registration require rigorous criteria. Antivenoms are complex biological products – antibodies produced by horses – which cannot be manufactured as generics. They require the use of venoms from local species, whose traceability should be guaranteed. Immunoglobulin purification and fragmentation should be performed using delicate processes, complying with standards set by WHO [7], and the application of quality control at every stage. The safety of antivenom should be favored as well as its effectiveness, especially since it is used in peripheral health centers, often poorly equipped and supplied. These characteristics explain the high price of antivenoms.
4. **The accessibility of antivenoms** should be ensured through appropriate funding, defined after anthropological investigations on the acceptability of the price by the affected population. An equalization of antivenom costs will involve the state budget, support from local governments, companies employing workers at risk (such as agribusiness corporations), and health insurance groups that are beginning to work in Africa.

Representatives from each country made a commitment to convey these recommendations to national health authorities and put in place the measures needed to achieve them before the next international conference on envenomation in Africa, which should happen in 2018.
